# Implementation of the WHO Pandemic Agreement 

**DOI:** 10.2471/BLT.25.294146

**Published:** 2025-09-16

**Authors:** WooJung Jon

**Affiliations:** a Graduate School of Future Strategy, Korea Advanced Institute of Science and Technology, 291 Daehak-ro, Yuseong-gu, Daejeon 34141, Republic of Korea.

The World Health Assembly adopted the historic World Health Organization (WHO) Pandemic Agreement by consensus on 20 May 2025.^1^ This treaty is only the second legally binding health treaty in WHO’s 77-year history, fundamentally reshaping the architecture of global pandemic preparedness and response. The treaty follows the 2003 Framework Convention on Tobacco Control, WHO’s first treaty negotiated under Article 19 of its Constitution. While the International Health Regulations of 2005 also impose legal obligations, they differ in legal nature, having been adopted under WHO Constitution Articles 21 and 22 as regulations rather than as a multilateral treaty.

The Pandemic Agreement incorporates a One Health approach in Article 5,^2^ recognizing that approximately 75% of emerging infectious diseases originate from animal sources. This integrated approach requires coordinated action across human health, animal health and environmental sectors to prevent zoonotic spillovers. Parties must establish multisectoral surveillance systems linking veterinary, wildlife and human health data (Article 4); strengthen laboratory capacity for cross-species pathogen detection (Article 6); develop joint response protocols between health and agricultural sectors (Article 5); and monitor high-risk environmental interfaces, such as wildlife markets and intensive farming operations (Article 4).^2^ For the first time, a binding pandemic instrument mandates comprehensive upstream prevention of zoonotic spillovers, moving beyond reactive measures to address pandemic risks at their source.^3^

## Vaccine equity

The international debate on equitable access during the coronavirus disease 2019 (COVID-19) was dominated by the pursuit of a patent waiver under the World Trade Organization’s (WTO) Agreement on Trade-Related Aspects of Intellectual Property Rights (TRIPS). On 17 June 2022, the WTO adopted a Ministerial Decision permitting the waiver of patents for COVID-19 vaccines without rights-holders’ consent.^4^ The decision was intended to enable low- and middle- income countries to more easily produce or export their own vaccines. However, the patent waiver’s effectiveness was severely limited. By November 2023, in low-income countries, vaccination coverage (of at least one dose) remained less than one third, considerably lower than in high-income countries, where it approached four fifths.^5^

## Technology transfer

The experiences of India and South Africa, leading advocates for the patent waiver, demonstrated that access to patents alone was insufficient to overcome production barriers.^6^ In South Africa, for instance, the case of Aspen Pharmacare demonstrated a different, yet equally critical, failure. After securing a license to produce and sell its own branded version of the Johnson & Johnson vaccine, its production lines remained inactive due to a lack of orders from African governments or global procurement mechanisms such as the COVID-19 Vaccines Global Access (COVAX) Facility.^7^ This outcome highlighted that even when patent and licensing barriers are overcome, local manufacturing can be undermined by market and procurement failures, another challenge the Pandemic Agreement’s comprehensive approach, including the Global Supply Chain and Logistics Network (Article 13), aims to solve. Advanced platforms like messenger ribonucleic acid (mRNA) vaccines depend on proprietary manufacturing processes, trade secrets and quality-control techniques that cannot be reverse-engineered from a patent document.^6^ The patent waiver failed to mandate the transfer of this essential know-how, rendering it largely ineffective for countries without pre-existing advanced manufacturing capacity.

This experience prompted a fundamental shift in global health governance, moving from a purely legalistic approach focused on patents to a pragmatic, operational one centred on building tangible production capabilities and technology transfer.^6^ Article 11 of the Pandemic Agreement embodies this shift, as it calls upon Parties and patent holders to share essential data, skills and proprietary information through WHO-led technology transfer hubs.^2^ By encouraging and facilitating transparent licensing, reasonable royalties and technical assistance, Article 11 aims to convert theoretical legal flexibilities into actionable opportunities for local manufacturing, addressing the critical know-how gap that the patent waiver left exposed.^8^ WHO’s evolving role in coordinating technology transfer represents a notable expansion of its traditional mandate.

## Implementation 

The central provision of the Pandemic Agreement is codified in Article 12, which establishes the Pathogen Access and Benefit-Sharing system. Under this system, signatories agree to provide WHO with timely access to pathogen genetic sequence data in exchange for a commitment from participating manufacturers to make 20% of their real-time production of pandemic-related health products available to WHO, with at least half of that amount (10% of total production) provided as donations and the remainder offered at affordable prices (Article 12.6(a)).^2^ For instance, if a total of 10 billion vaccine doses were produced during a pandemic, this provision would allocate about 2 billion doses to WHO (with roughly 1 billion of those as donations). This mechanism is designed to prevent a recurrence of the hoarding and inequitable distribution that defined the early COVID-19 response.

However, the Agreement’s entry into force faces an important procedural bottleneck that threatens to delay its implementation. While the framework was adopted in May 2025, the specific operational modalities of the pathogen access and benefit-sharing system were deferred to a separate, legally binding annex. This annex, which will define critical terms such as affordable prices and outline the logistics of the 20% allocation, is scheduled to be negotiated and presented for consideration at the seventy-ninth World Health Assembly in May 2026.

Article 31 of the Pandemic Agreement stipulates that it will only be open for signature after the pathogen access and benefit-sharing annex is adopted.^2^ This article creates a critical dependency: without a successfully negotiated annex, the process of ratification cannot even begin. The Agreement requires ratification by at least 60 Parties to enter into force (Article 33),^1^ a process that can only commence after the complex and politically sensitive details of the benefit-sharing mechanism are finalized (Article 31).^2^ This 2025–2026 negotiation period therefore represents the most important hurdle to the Pandemic Agreement’s viability. Failure to reach consensus on the annex would render the historic 2025 adoption a symbolic but ultimately hollow victory.^9^

## Fractures and challenges

Even if the annex is successfully negotiated, the Agreement’s effectiveness will be severely tested by geopolitical fractures. The nonparticipation of the United States of America, citing concerns over the binding nature of technology transfer provisions, sovereignty, intellectual property obligations, financial commitments and impacts on pharmaceutical innovation incentives, and the abstention of 11 key countries reflect deep geopolitical divisions. The nonparticipation of the United States poses a notable threat,^10^ as the country is home to many of the world’s leading pharmaceutical innovators, including Eli Lilly, Johnson & Johnson, Pfizer, AbbVie and Merck. The nonparticipation of these United States-based companies, which operate some of the world’s most extensive global supply chains, effectively decouples a primary engine of biopharmaceutical research and development from the pathogen access and benefit-sharing system, and creates a major structural void in the global supply chain and logistics network under Article 13 of the Pandemic Agreement.

The abstentions of other governments introduce further layers of complexity. The 11 abstaining countries, including Israel, Italy and the Russian Federation, expressed reservations about the system’s mandatory benefit-sharing requirements and concerns over national security implications of pathogen data sharing. Italy’s absence from the agreement creates considerable jurisdictional and logistical uncertainty. Italy is home to major domestic firms such as Menarini and Alfasigma, and also serves as a crucial manufacturing hub for multinational corporations such as Pfizer, Novartis and AbbVie.^11^ This nonparticipation could potentially disrupt key nodes in the European supply chain. Similarly, Israel’s abstention jeopardizes the supply of affordable, high-volume generic medicines and essential active pharmaceutical ingredients that are critical for an equitable response in low- and middle-income countries, because Israel is home to Teva Pharmaceuticals, a leading producer in this sector. Finally, the Russian Federation’s abstention, coupled with its growing domestic biotech and pharmaceutical manufacturing capacity through companies like BIOCAD and R-Pharm, risks the formation of a parallel, non-integrated pandemic response bloc that could undermine WHO’s central coordinating role and fragment global supply chains.^12^

## Conclusion

The WHO Pandemic Agreement, with its mechanisms to encourage technology transfer and benefit sharing, represents a monumental achievement in the evolution of global health law. The Agreement rightly identifies the transfer of practical know-how, not just patent rights, as the key to achieving genuine equity in pandemic response. However, the potential codified in its text is severely challenged by the realities of its implementation environment. The procedural dependency on the 2026 annex negotiation creates a critical point of delay. More profoundly, the geopolitical fractures marked by the nonparticipation and abstention of key countries threaten to undermine its core principles by fragmenting private sector engagement and global supply chains. The capacity of the Parties to overcome this implementation challenge and to bridge the divide between the Pandemic Agreement’s ambitious goals and a fragmented geopolitical landscape will ultimately determine whether the Agreement can truly reshape the global response to future pandemics.

Future research should examine how enforcement mechanisms can be strengthened without compromising sovereignty, and how the Agreement’s provisions might adapt to emerging technologies such as artificial intelligence-driven drug discovery platforms and mRNA platform technologies for rapid vaccine development.
